# Using hypnotic suggestion in the rehabilitation of working memory capacity after acquired brain injury: study protocol for a randomized controlled trial

**DOI:** 10.1186/s13063-023-07867-z

**Published:** 2024-01-02

**Authors:** Line Sophie Eide, Per-Ola Rike, Silje Endresen Reme, Hildegun Snekkevik, Stephan Rossner, Gunnar Rosen, Jonas Kristoffer Lindeløv, Marianne Løvstad

**Affiliations:** 1 Sunnaas Rehabilitation Hospital, Nesodden, Norway; 2 University of Oslo (UiO), Oslo, Norway; 3 Norwegian Society of Clinical Evidence-Based Hypnosis (NEKEH), Oslo, Norway; 4 Molde Hospital, Molde, Norway; 5 Aalborg University (AAU), Aalborg, Denmark

**Keywords:** Acquired brain injury, Cognitive rehabilitation, Clinical hypnosis, Medical hypnosis, Working memory capacity, Self-efficacy, Clinically relevant changes, Everyday functioning

## Abstract

**Objectives:**

Establishment of effective evidence-based interventions in rehabilitation of working memory (WM) deficits after acquired brain injury (ABI) is sorely needed. Despite robust evidence for the efficiency of clinical hypnosis in a wide range of clinical conditions, and improved understanding of mechanisms underlying its effects, the potential of clinical hypnosis in cognitive rehabilitation is underexplored. A recent study has shown large effects of hypnotic suggestion on WM capacity following ABI. This randomized controlled trial aims to evaluate and explore the replicability of these findings and examine the generalization of treatment effects. The study will also explore possible mechanisms of change.

**Methods:**

Ninety patients will be recruited from the Sunnaas Rehabilitation Hospital. Inclusion criteria are nonprogressive ABI, minimum 12-month post-injury, ongoing WM deficits, and age between 18 and 67 years. Patients will be randomized to either (a) an intervention group receiving four weekly 1-h sessions with induction and hypnosis, (b) an active control group receiving four weekly 1-h sessions of induction and mindfulness, or (c) a passive control group without intervention. The targeted procedure consists of suggestions about enhancing WM functions, for example through the instantiation of preinjury WM capacity in the present using age regression or through visualizations of brain plasticity. The non-targeted suggestions contain no explicit mention of ABI- or WM-related abilities. Each participant will be assessed at baseline, immediately after intervention, and 6 months after baseline. The primary outcome is the WM index from WAIS-IV and self- and informant-reported WM subscale from BRIEF-A, a questionnaire exploring executive functioning in everyday life. Secondary outcomes include a cognitive composite score derived from tests measuring processing speed, executive functions, learning capacity and memory, and self-reported measures of emotional distress, quality of life, and community integration. Exploratory measures include self-rated ABI and WM-related self-efficacy.

**Discussion:**

Rehabilitation of impaired WM after ABI has hitherto yielded limited transfer effects beyond the training material, i.e., improvement effects on everyday WM capacity, and clinical trials of new interventions are thus warranted. Long-standing empirical evidence demonstrates that hypnosis is an effective therapeutic technique in a wide range of conditions, and recent exploratory research has suggested a high efficacy of hypnosis in improving WM capacity in patients with ABI. However, these extraordinary findings need replication in studies applying scientifically rigorous designs. If successful, our ambition is to provide recommendations and materials to implement hypnotic suggestion as an adjunct treatment following ABI. Study findings may inform future studies exploring the use of clinical hypnosis in other areas of rehabilitation, such as mild TBI, and in other neurological conditions where WM deficit is prominent.

**Trial registration:**

ClinicalTrials.gov NCT05287542. Registered on March 2022

**Protocol version:**

Protocol version 2.0, December 2023.

**Supplementary Information:**

The online version contains supplementary material available at 10.1186/s13063-023-07867-z.

## Introduction

### Background

Working memory (WM) is regarded as the “sketchpad of conscious thought” [1], a cognitive capacity that holds and manipulates a limited amount of information for a short time in order to produce a response [2]. Multiple brain regions are involved in WM, and WM impairment is one of the most prevalent symptoms after acquired brain injury (ABI) [3], regardless of lesion etiology, localization, and severity [4]. WM is a central cognitive ability upon which rehabilitation for other functions depends and is a predictor for rehabilitation outcome, activity of daily living (ADL), physical rehabilitation, need for community services after discharge from hospital, community participation, and occupational status [5–10]. Rehabilitation efforts to improve high-level cognitive functioning following ABI have yielded limited clinically relevant effects so far [11–13]. Despite repeated administrations, reviews of biological interventions such as pharmaceuticals [14], noninvasive brain stimulation [15], physical exercise [16], and nutrition [17] show effect sizes in the zero to moderate range. Mindfulness can potentially reduce ABI-related fatigue. However, no significant effect on WM is found [18]. Treatment of WM through computerized training programs is widespread, regardless of systematic reviews reporting small to no clinically WM-relevant changes [19]. A comprehensive review of the brain-training literature reported no evidence for improvements on “far-transfer” cognitive abilities [19]. In other words, the improvements failed to generalize to other capacity-dependent activities [20]. Previous WM rehabilitation efforts have typically focused on “bottom-up” training programs that aim to restore function through repeated drills and graded exercise [12], where transfer of effects to untrained functional domains has not been documented.

In contrast to the train and drill bottom-up approach, the use of clinical hypnoses represents a “top-down” approach and has been suggested by Lindeløv and colleagues [4], to improve WM performance and to potentially enable generalization to other contexts.

The hypnotic state is a condition of openness to suggestions so that they may be used to elicit changes in a diverse array of psychological and bodily functions [21]. Reviews of hypnotic treatment in psychology [22] and medicine [23] are impressive, with demonstrated efficacy for anxiety [24], depression [25, 26], chronic pain [27], and headache [28] as an adjunct or alternative to anesthesia during surgery [29], in neurorehabilitation for motor disorders [30, 31], pain and vertigo [32], and aphasia [33]. Recent publications have made advancements in identifying the neural correlates underlying the hypnotic state [34]. Recently, hypnotic suggestions were found to enhance updating in WM in healthy adults, which was associated with changes in event-related potentials (ERP) in WM-related regions of the brain [35], indicating that hypnotic suggestions can be potent in altering cognitive functions*.* A few hypnosis studies have been conducted aiming at enhancing cognitive and psychological functions after ABI [36–38]. However, previous studies suffer from one or more major methodological weaknesses, such as a lack of control groups, lack of detailed descriptions of participants, randomization, blinding of testers, statistical procedures, and suggestions used during hypnosis.

The only randomized controlled trial (RCT) to date to include hypnosis in WM rehabilitation was performed by Lindeløv and colleagues [4]. This RCT included 68 participants with ABI across 3 treatment arms: an intervention group, an active control group, and a passive control group. The intervention included techniques such as age regression and visualizations of brain plasticity with suggestions about enhancing WM functions through the instantiation of preinjury WM ability in the present. The non-targeted suggestions contained no explicit mention of ABI or WM-related abilities but was otherwise matched in length and procedure, thus serving as an active control. Contrary to previous research, this study reported large effect sizes after only four 1-h sessions in favor of the intervention group compared to both active controls (Bayes factors of 342 and 37.5 on the two aggregate outcome measures) and the passive control group (Bayes factor = 1.7 × 10^13^). The long-term effect remained at approximately 6-week follow-up (Bayes factors = 7.1 and 1.3 in favor of no change). The outcome measures were neuropsychological tests, i.e., the WM index from WAIS-IV and Trail Making Test A and B. The authors suggest that hypnosis can improve WM following ABI, and that the speed and magnitude of the improvements indicate that there may be a potential for releasing residual cognitive capacity after ABI rather than “building” it anew. However, this study lacked medical data such as brain injury characteristics; it included only a few outcome measures (i.e., neuropsychological tests) and, most importantly, no measures of everyday WM capacity. Furthermore, Lindeløv and colleagues did not explore the potential underlying mechanisms associated with improved WM among the participants.

Self-efficacy theory asserts that an individual’s beliefs in personal competency predict actual performance [24], and ABI survivors often display negative self-expectancies and show lower WM self-efficacy than healthy controls [25]. Self-efficacy influences coping style and quality of life, satisfaction with functioning [26], social participation [27], functional independence [28], and community integration [29] after ABI. Thus, changed expectations about the possibility of dealing with the consequences of brain injury, i.e., improved self-efficacy, are a potential candidate in mediating generalization effects in cognitive rehabilitation [26]. Hypnosis seems to be particularly well positioned to enhance self-efficacy [30] and change expectations of psychological and behavioral outcomes in order to build confidence in one’s ability to cope with or solve problems [31, 32, 39, 40]. A person’s brain injury expectations may influence their cognitive performances through mechanisms similar to the nocebo effect [41]. Nocebo is a self-fulfilling prophecy where adverse effects are produced by expectations [42, 43]. The Cognitive Activation Theory of Stress (CATS) [44] suggests that expectancies regulate bodily stress responses [45]. This is relevant to the current study given the established negative influence of stress-related cortisol on WM capacity [46]. Specifically, expectations about coping (i.e., self-efficacy) may reduce the stress response, while expectations of helplessness or hopelessness may sustain it [44, 47]. This study will test the hypothesis that hypnosis in WM rehabilitation exerts its effect partly through changes in WM self-efficacy.

### Objectives and hypothesis

The main objective of this study is to explore the effect of hypnotic suggestion on WM capacity in adults with ABI. The following three research questions will be explored:

1. *Are the strong effect of hypnotic suggestion on WM performance in ABI patients found by Lindeløv et al. (2017) replicable?*

Our hypothesis is that the direction and the magnitude of the effects on WM found by Lindeløv and colleagues after four treatment sessions will be replicated. Given the large changes seen in Lindeløv et al.’s study, even weaker results are of clinical interest.

2. *Does hypnotic suggestions improve everyday WM, everyday functioning and participation, emotional status, and quality of life in ABI patients?*

The intervention is hypothesized to improve WM performance through “top-down” processes, which will allow improved WM capacity to be applied across diverse tasks and contexts. Thus, we expect to see treatment-related change in the domains of daily life, emotional status, and quality of life.

3. *Does increased self-efficacy predict the effect of hypnosis on WM?*

We hypothesize that changes in self-efficacy predict WM improvements following hypnosis.

## Methods

### Trial design

The study is organized as a randomized controlled superiority trial with parallel group design and three arms including (1) an intervention group, (2) an active control group, and (3) a passive control group. Participants will be randomly allocated in a 1:1:1 ratio to either of the three groups. The intervention group will receive four weekly 1-h sessions with hypnosis treatment (induction + hypnotic suggestion), while the active control group will receive four weekly 1-h sessions of non-targeted hypnotic suggestions (induction + mindfulness-based instructions) to factor out nonspecific treatment effects. The passive control group will be assessed at the same time points as the other two groups to isolate retest effects. Each participant will be assessed three times: (1) at baseline (T1), (2) immediately after the intervention which is 5 weeks post baseline (T2), and (3) 25 weeks after baseline (T3, follow-up). The study will follow the CONSORT statement facilitating complete and transparent reporting and aiding critical appraisal and interpretation (http://www.consort-statement.org/). This study expands the design of Lindeløv et al.’s study through a larger number of participants, the inclusion of far-transfer outcome measures, and the exploration of potential underlying mechanisms of treatment effects. Because this study is performed in collaboration with the principal investigator of the original study [4], we rely on learnings from that study rather than conducting a small-scale pre-study pilot and/or feasibility trial. The intervention will be based on the hypnosis scripts by Lindeløv et al. translated from Danish to Norwegian [4].

### Study setting and participants

Eligible participants are recruited at the department of cognitive rehabilitation at Sunnaas Rehabilitation Hospital (Bjørnemyrveien 11, 1453 Bjørnemyr, Norway). The department discharge approximately 370 patients a year making it realistic to reach the target sample size. Assessments and interventions will be conducted at the outpatient clinic at the Sunnaas Rehabilitation Hospital. The therapist will collect signed written informed consent forms before baseline assessments.

### Eligibility criteria (10)

#### Inclusion and exclusion criteria

Inclusion criteria are a documented nonprogressive ABI, minimum 12-month post-injury, ongoing WM deficits (by self-report and/or neuropsychological assessment), and age between 18 and 67 years. Exclusion criteria are severe mental illness (e.g., diagnosed major depressive disorder, schizophrenia, or bipolar disorder), progressive neurologic disease, and/or ongoing ICD-10 diagnosis of substance use disorder or lack of Norwegian language skills. Participants will be discouraged from participating in intensive in-patient cognitive rehabilitation while included in the trial but will not ask them to refrain from ordinary long-term community-based treatment such as physiotherapy and/or occupational therapy. Service provision during the trial will be documented.

### Included measures

**Fig. 1:**
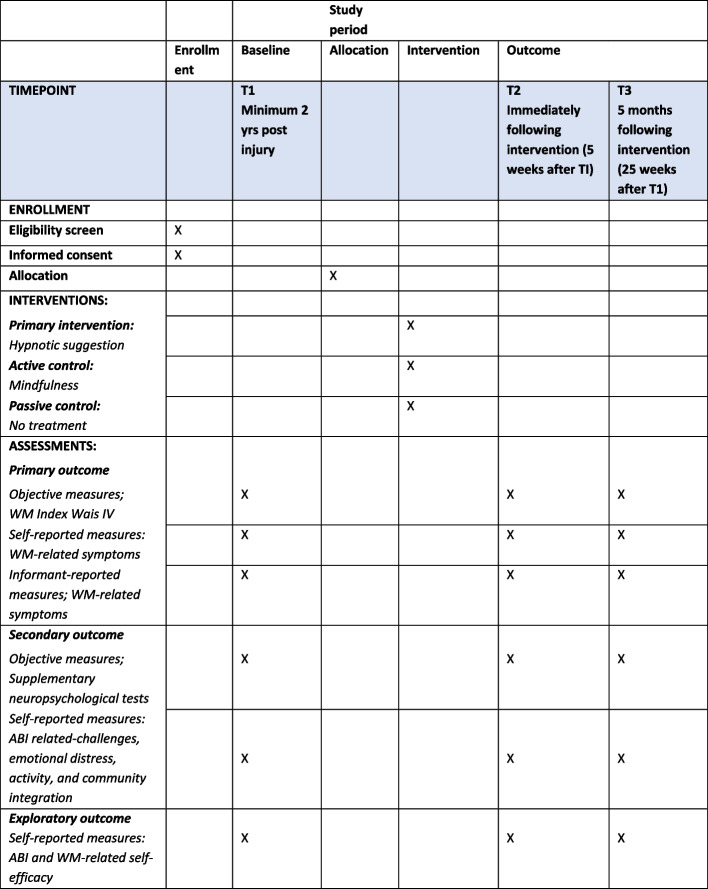
Standard Protocol Items: Recommended for Intervention Trials (SPIRIT)

Three sources of data will be collected: (1) demographic and medical data, (2) performance-based neuropsychological measures, and (3) subjective ratings of WM, emotional distress (anxiety and depression), quality of life, activity, and community integration (Fig. 1).

### Patient characteristics

Sociodemographic variables will include age, gender, marital status, children, living conditions, years of education, profession, and study or employment status. Medical variables include preexisting comorbidity, substance use, concurrent somatic and psychiatric diseases and injury characteristics of interest time since injury, etiology, and results of radiological examinations for lesion characterization.

### Interventions

The treatment protocol is identical to the study that is being replicated. During the first phase of the study, the treatment group and the active control group will receive identical procedures, including welcoming, hypnotic inductions, hypnotic re-alerting, and farewell. The groups differ in between the hypnotic induction and hypnotic re-alerting. The targeted procedure consists of suggestions about enhancing WM functions through the instantiation of preinjury WM ability in the present using age regression and visualizations of brain plasticity. The non-targeted suggestions contain no explicit mention of brain injury or WM-related abilities and thus serve as an active control, i.e., to isolate the ‘targetedness’ of suggestion as the independent variable by factoring out other influences from placebo, retest effects, etc. These non-targeted suggestions are borrowed from mindfulness meditation practices, involving body and thought awareness as they have demonstrated no or small effects on cognitive abilities in participants with ABI [48, 49]. All sessions will last for about 1 h, and the patients are invited to either sit in a comfortable chair or lay down on a bench during the procedures. We have access to the full treatment manual used by Lindeløv et al. in Danish. The manual has been translated into Norwegian. Throughout the spring of 2021, we tested the manual with the active treatment condition with two user representatives at Sunnaas Rehabilitation Hospital. This has given us the opportunity for closer collaboration with users regarding feasibility and adherence to intervention protocol.

To secure compliance to the protocol, regular supervision is provided to the therapist conducting the intervention. There are few side effects of hypnosis, and most people report well-being after hypnotic intervention. Participants are informed about the harmless nature of hypnosis, but also that if they wish to end the session for any reason, treatment will be immediately aborted. The therapist registers any occurrence of uncomfortableness or adverse effects of the treatment. Participants will further be informed that this is an experimental study with unknown effects. Participation is not time-consuming and will not be at the expense of other treatment.

### Outcomes

The main objective outcome measure is the WM index from WAIS-IV, and the main subjective outcome is the self- and informant-reported WM subscale from the Behavior Rating Inventory of Executive Function–Adult version (BRIEF-A) [50]. Secondary objective outcome measures include neuropsychological tests measuring (1) processing speed (Trail Making Test Part A (TMTA)), Color Word Interference Test (CWIT) parts 1 and 2 from Delis-Kaplan Executive Function System (D-KEFS), and Digit Symbol Test from Wechsler Adult Intelligence Scale 4th Edition (WAIS-IV) [51], (2) executive functions (CWIT parts 3 and 4 from D-KEFS [52] and Trail Making Part B (TMT B)), and (3) learning capacity and memory (California Verbal Learning Test (CVLT-II)). Secondary subjective outcomes include the following: (1) emotional distress (The Hopkins Symptom Checklist (HSCL)) [53], (2) quality of life (QOLIBRI) [54], (3) community integration (Patient Competency Rating Scale (PCRS) [55], and (4) everyday participation (The Participation Assessment with Recombined Tools-Objective (Part-O)). Changes in self-efficacy will be measures with the Traumatic Brain Injury Self-Efficacy Questionnaire (TBI-SE) [56] and the Memory Self-Efficacy Questionnaire (MSEQ) [57]. All measures will be applied at all three time-points (Table 1).

**Table 1:** Overview over measures included at baseline and outcome assessments

			Baseline (T1)	Follow-up, 5 weeks (T2)	Follow-up 25 weeks (T3)
1a. Main objective outcome measure					
	*Working memory*	WAIS-IV Working Memory Index (digit span, letter-number sequencing, and mental arithmetic)	X	X	X
1b. Main subjective outcome measure					
	*Working memory*	Self and informant rating of the working memory scale in BRIEF-A	X	X	X
2a. Secondary objective outcome measures					
2b.	Secondary subjective outcome measures	Trail making A+B, D-KEFS CWIT 1-4, CVLT-II, WAIS-IV coding	X	X	X
	*Emotional distress*	The Hopkins Symptom Checklist (HSCL-25)	X	X	X
	*Activities of daily living*	Patient Competency Rating Scale (PCRS)	X	X	X
	*Quality of life*	Quality of Life after Brain Injury (QOLIBRI)	X	X	X
	*Community integration*	Participation Assessment with Recombined Tools-Objective (PART-O)	X	X	X
3. *Self-efficacy*					
		The TBI Self-Efficacy Questionnaire (TBI-SE) and Memory Self-Efficacy Questionnaire (MSEQ)	X	X	X

### Participant timeline

**Fig. 2:**
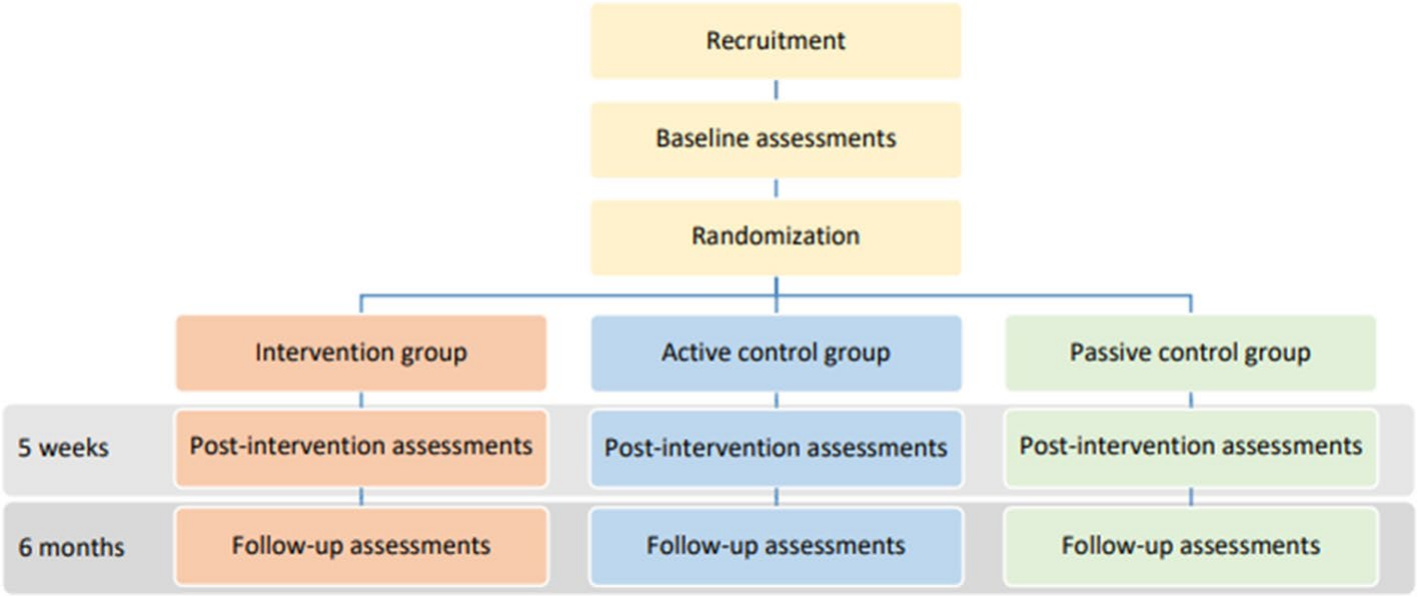
Trial design

A study flowchart is provided in Fig. 2.

### Sample size and power calculations

Although Lindeløv et al. [4] found large treatment effects, their novel and unreplicated nature causes us to design a study that will also be informative with medium effect sizes. We set a sample size so that we can detect clinically meaningful changes of 0.5 SD on the primary outcome measures, i.e., the WAIS-IV Working Memory Index and BRIEF-A Working Memory Scale. Computed in G*Power, the group × time within-between interaction effect would require 22 completers in each group for a medium-sized effect (*ηp*^2^ = 0.07), 1% significance level (due to corrections for multiple testing), and a power of 90%. Allowing for a 20–25% drop-out rate brings the required sample to 30 participants per group, i.e., a total of *n* = 90. The success criterion in this study is thus defined as medium effect sizes (*ηp*^2^ = 0.07).

### Randomization and blinding

Eligible patients will be identified by the study PI (author L. S. E.) from the department of cognitive rehabilitation (KReSS) at Sunnaas Rehabilitation Hospital. After baseline assessment, participants will be randomly allocated to either of the three groups. The randomization sequence will be generated electronically in STATA by an independent statistician.

The allocation sequence will be stored in a database that can only be accessed by the study’s principal investigator. It will not be possible to blind neither the participants nor the PhD candidate who will conduct the hypnotic suggestions. Outcome assessments will, however, be blinded in that a person external to the study will perform these without knowledge of group allocation. Our research group has extensive experience in managing blinded clinical trials, and we are confident that this can be conducted in a truly blinded way without contamination. Data analysis will also be blinded in that fake ID numbers will be assigned to participants in the final database, ensuring that the statistical analysis is performed without knowledge of true group content until after the analyses are finalized.

### Dropout and data retention

Participants that want to exit the study can do so at any given time by informing the study PI or the therapist providing the treatment. Their names and contact information are provided in the consent form. In case of dropout, the participant is asked if they are willing to report why they want to exit the study. If information regarding reason for exit of the study is provided, the information is registered on a dedicated drop-out sheet. Unless the participant requires all their data deleted, data is still included in the intention-to-treat (ITT) analyses.

### Statistical methods

Intention-to-treat (ITT) analysis will be applied, using data from all randomized participants, regardless of whether they complete the intervention. Descriptive statistics will be reported for sample description and outcome measures. The effect of the intervention will be assessed by linear mixed-effect models fitting the primary and secondary continuous outcome variables to account for repeated measurements by patients. Time (T1, T2, and T3) and time-by-treatment interaction will be used as fixed effects in these models. The main effect of group will be included to control for potential baseline differences. Based on the linear mixed effect models, we will estimate mean values with 95% confidence intervals (CI) for the three time points (T1, T2, and T3) for each treatment group. We will also estimate the mean between-group changes from T1 to T3. These models account for missing data on individual time points, thus obviating the need to impute missing values. We will preregister the analytic strategy. The role of self-efficacy on the effect of hypnosis will be explored with linear regression models. No interim analyses will be performed.

### Ethical issues

The intervention will be carried out in accordance with guidelines from the Norwegian Society of Clinical Evidence-Based Hypnosis (NFKEH) by a clinical psychologist educated in hypnotherapy. Written, informed consent to participate will be obtained from all participants. The study will be conducted in accordance with the Declaration of Helsinki and the Vancouver recommendations for the conduct, reporting, editing, and publication of scholarly work in medical journals. The study has been approved by the Regional Committee for Medical and Health Research Ethics (approval number 216495) and the Norwegian Center for Research Data (NSD) (approval number 291031). The research is registered and made public at the Clinical Trials and Open Science Framework.

### Dissemination plans

The study results will be presented at Norwegian and international conferences in neurology, brain injury, and rehabilitation and in various arenas in which the Norwegian Union of Stroke (NFS) is active. The study results will be disseminated in Norwegian academic environments, and popular scientific presentations will be given. The results will also be communicated to the public through commercial channels like newspaper articles and social media. A dissemination strategy will be established in collaboration with the user panel who will actively participate in disseminating the findings and their relevance for patients through their channels. Given the close collaboration between Sunnaas Rehabilitation Hospital and other rehabilitation centers in Norway, we are well-positioned to communicate findings to other hospitals and stakeholders. Moreover, the Norwegian Society of Clinical Evidence-Based Hypnosis (NFKEH) provides education in hypnosis to health personnel subjected by the Norwegian Health Personnel Act. Since the head of NFKEH participates in this study, clinical hypnosis education of neurorehabilitation professionals will be provided.

## Discussion

The potential impact of applying hypnosis in cognitive rehabilitation is substantial. Deficits in WM are one of the most common challenges after ABI [3] and play a critical role in post-injury outcome [29]. Still, state-of-the-art approaches within WM rehabilitation have not been able to produce clinically relevant effects on real-life functioning for the affected patients [19]. In addition, today’s ABI rehabilitation is often time-consuming and requires hospitalization. As described by, e.g., Cicerone and colleagues [12], there is a need for more effective cognitive rehabilitation methods. Long-standing empirical evidence demonstrates that hypnosis is an effective therapeutic technique for a wide range of conditions [23], including in altering cognitive functions and improving WM in healthy adults. These findings are supported by changes seen in functional MRI [58] and event-related potentials [35]. Recent explorative research has suggested a promising efficacy of hypnosis in improving WM capacity in patients with ABI [4]. However, these findings are in need of replication in studies applying scientifically rigorous designs, and there is a need to understand potential underlying mechanisms of change.

Building on the strong results in the Danish single trial, we will aim at replication and expansion of the study in a Norwegian context. The initial study will be expanded in terms of the number of participants, injury characteristics will be included, and outcome measures of relevance to real-life functioning and possible underlying mechanisms of change will be explored. Self-efficacy is generalizable by nature (self-efficacy gained from mastery experiences in one situation generalizes to others); thus, WM rehabilitation effects are expected to generalize and transfer to other domains in daily life.

If previous findings are replicated, and transfer effects documented, patients may have improved WM capacity, including everyday WM, improved activities of daily living, self-efficacy, psychological well-being, and quality of life. These factors are critical to independence and may in turn affect the need for community support, community integration, and return to work. The intervention is manual based and short (four sessions) and is conducted in an outpatient clinic. Hypnotic interventions are typically brief, cost-effective, suitable for both in- and outpatients, for adolescents and adults, and can be learned easily; suggestions can be administered either by another person (hetero-hypnosis) or self-administered (self-hypnosis), adding portability to the mix of benefits. As the study that is being replicated is the only one to test hypnosis in rehabilitation of WM, a lack of replication, i.e., null findings, would also be highly informative to the field. Regardless of outcome, this study can provide valuable insights to other research groups and inform future studies. Study findings may inform future studies exploring the use of clinical hypnosis in other areas of rehabilitation, such as mild TBI (i.e., post-concussion symptoms), and in other neurological conditions where WM deficit is prominent, such as multiple sclerosis and Parkinson’s disease. A next step could also be to explore potential effects beyond the domain of WM, as studies have shown that self-efficacy is associated with mental health [27], quality of life [26], functional independence [28], and community integration [29]. This study thus has the potential to influence how we understand the clinical significance of psychological factors, like self-efficacy, on functioning after ABI. If successful, our ambition is to provide recommendations and materials to implement hypnotic suggestion as an adjunct treatment following ABI in Norwegian rehabilitation clinics.

### Trial status

The study was approved by the Regional Committee for Medical and Health Research Ethics in May 2021 and funded by Foundation Dam in December 2021. Recruitment for the RCT began at Sunnaas Rehabilitation Hospital in October 2022 and will end when we have enrolled the estimated sample size of 90, estimated June/July 2024.

### Plans for communicating important protocol modifications

Protocol modifications of importance will be reported to the Data Protection Office at Sunnaas Hospital, and amendments will be made to the trial registry (ClinicalTrials.gov) and Open Science Framework.

### Supplementary Information

**Additional file 1.** Spirit checklist [59].

## Data Availability

Data will be stored electronically on a secure research server at Sunnaas Rehabilitation Hospital. The data will be kept for 5 years after the end of the project for control reasons. All principal investigators will be given access to the cleaned data sets. Access to data is regulated by Norwegian laws regarding data protection and research ethics.

## Abbreviations

ABI: Acquired brain injury
WM: Working memory
ADL: Activity of daily living
RCT: Randomized controlled trial
CATS: Cognitive activation theory of stress
NFKEH: The Norwegian Association of Clinical Evidence-based Hypnosis
ITT: Intention to treat
WAIS-IV: Wechsler Adult Intelligence Scale–Fourth Edition
TMTA: Trail Making Test Part A
TMTB: Trail Making Test Part B
CVLT-II: California Verbal Learning Test–Second Edition
BRIEF-A: Behavior Rating Inventory of Executive Function–Adult version
TBI-SE: The Traumatic Brain Injury Self-Efficacy Questionnaire
MSEQ: The Memory Self-Efficacy Questionnaire
HSCL: The Hopkins Symptom Checklist
QOLIBRI: Quality of life
PCRS: Patient Competency Rating Scale
Part-O: The Participation Assessment with Recombined Tools-Objective
